# Discrete photoelectrodes with dyes having different absorption wavelengths for efficient cobalt-based tandem dye-sensitised solar cells

**DOI:** 10.1038/s41598-017-02480-y

**Published:** 2017-05-23

**Authors:** Phuong Ho, Suresh Thogiti, Yong Hui Lee, Jae Hong Kim

**Affiliations:** School of Chemical Engineering, Yeungnam University, 214-1, Dae-dong, Gyeongsan-si, Gyeongsangbuk-do, 712-749 Republic of Korea

## Abstract

A pn-tandem dye-sensitised solar cell (pn-DSSC) employing a set of sensitisers with complementary absorption spectra and a less-corrosive cobalt-based electrolyte is presented. We applied three organic sensitisers (denoted C343, DCBZ, and SQ) featuring different absorption wavelengths for the p-DSSC, while keeping the n-DSSC sensitiser (denoted DCA10CN2) constant. Characterisation of the Co^+2/+3^-based DSSC devices revealed that SQ dye, with a longer absorption wavelength, showed broader spectra and increased photocurrent activity in the visible and near-infrared region compared to the other two devices with C343 and DCBZ in the pn-DSSCs. As a result, the short-circuit current density increased significantly to 4.00 mA cm^−2^, and the devices displayed overall power conversion efficiencies of as high as 1.41%, which is comparable to that of the best pn-DSSCs in the literature. Our results demonstrate that complementary absorption between the two photoelectrodes is important for enhancing the photovoltaic performance of pn-DSSCs.

## Introduction

**Figure 1:**
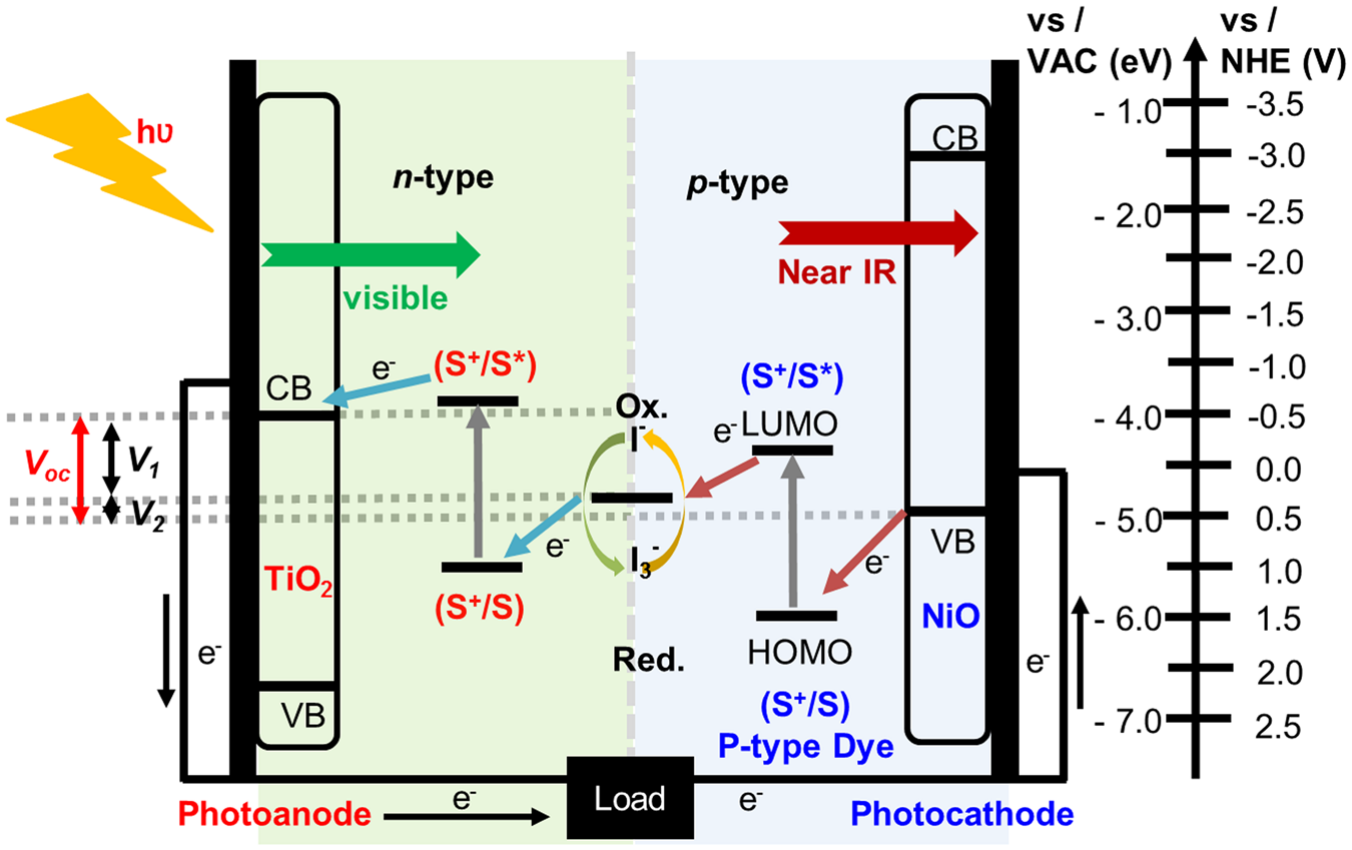
Schematic representation of tandem pn-DSSC.

Dye-sensitised solar cells (DSSCs) incorporating mesoporous-network-based photoelectrodes are often suggested as a low-cost replacement for conventional silicon-based solar cells^1^. However, their power conversion efficiency (PCE) to date has reached only 13%, as compared to 25% for crystalline-silicon-based solar cells^2, 3^. Conventional DSSCs are based on photoanodes, where the photocurrent results from excited electron transfer into the conduction band of TiO_2_. The dye molecules typically employed in these devices can harvest only the visible or the near-infrared (IR) part of the solar spectrum, resulting in the loss of the IR or visible part, respectively. If these lost IR/visible photons could be properly used, extreme improvement in the PCE could be predicted. Although the use of near-IR sensitisers or co-sensitiser-based DSSCs compensates for the light-harvesting limitations, these methods cannot improve the theoretical upper limit because they are still single DSSCs^4, 5^. On the other hand, the application of tandem architecture is a novel strategy considered to enrich the light harvesting ability of DSSCs^6–9^. Such devices are generally fabricated using two separate photoelectrodes. A dye-coated photocathode (p-type) with absorption complementary to that of the photoanode (n-type) would enable fabrication of pn-type tandem cells, where the Pt-based counter electrode found in conventional DSSCs (n-type) is replaced with a dye-sensitised p-type semiconductor^10, 11^. This allows more photons to be converted more efficiently. This is a promising way to increase the spectral coverage and improve the PCE of DSSCs (Fig. 1). Previous results had demonstrated that the light harvesting and performance of DSSCs can be boosted by using a tandem-based architecture^12–17^.

To date, more than 90 different kinds of sensitisers have been evaluated as sensitisers in p-type DSSCs. But, until recently, commercially available C343 sensitized NiO remained the ‘unofficial standard’ for p-DSSCs^18–28^. On the other hand, double branched dyes have received great attention in n- and p-type DSSCs and obtained improved PCE compared to the corresponding single branched dye^29–34^. The red absorbing cationic indolium unit has recently been utilised for enabling the dye to absorb the long wavelength visible light in p-DSSCs^35^. In this direction, Park *et al*. reported a novel carbazole-based double branched dye DCBZ that contained red absorbing cationic indolium acceptor, and obtained enhanced PCE in p-DSSCs compared to corresponding single branched dye and reference C343. Several groups reported on squaraine-based sensitisers applied for photocathode in p-DSSCs^14, 36–39^, for example, the p-type devices using squaraine-arylamine dyes achieved efficiencies of 0.053% and 0.113% for the dyes containing one and two anchoring groups, respectively^36^. Furthermore, Warnan *et al*. employed iodo-squaraine (SQ), a squaraine-perylene monoimide (SQ-PMI) dyad, and a squaraine-perylene monoimide-naphthalene diimide (SQ-PMI-NDI) triad on NiO photocathode for p-DSSCs using iodine-based redox couple (I^−^/I_3_^−^) or Co-based redox couple^37^. Recently, Powar *et al*. reported the application of SQ dye as sensitiser for photocathode and PMI-6T-TPA for photoanode with the use of thiolate/disulfide-based electrolytes giving PCE of 0.51% for p-DSSC and 1.3% for pn-DSSC^14^. More recently, Bonomo *et al*. synthesised three symmetrical SQ dyes (VG1, VG10 and VG11) and employed as sensitisers for p-DSSCs and dye with a dicyano-vinyl substituent on the central SQ ring (VG11) achieved highest PCE of 0.043%^38^. Clearly, compared to the number of reports on p-type DSSCs, quite few tandem pn-DSSCs have been documented. The first pn-DSSC, which had a PCE of 0.39%, was reported by He *et al*. in 2000^6^, and the highest PCE to date is 1.91% under AM 1.5 illumination^12^. In 2014, Shao *et al*. developed a novel polymer-based photocathode for a pn-DSSC and realised a PCE of 1.30%^13^. A pn-DSSC with a novel D-π-A sensitiser for the photocathode was recently developed and showed a PCE of 1.7%^11^. Since the first pn-DSSCs were reported, different types of p-type semiconductors have been reported^40–44^; among them, dye-sensitised NiO photocathodes are most commonly studied for p-DSSCs^10, 25, 40–53^. However, the PCE of tandem pn-DSSCs remains lower than that of conventional DSSCs at present.

The I^−^/I_3_^−^ is the most commonly used redox electrolyte for both n-type and p-type DSSCs. The complicated redox reactions, high corrosiveness, and strong light absorption at wavelengths below 500 nm of this electrolyte system have directed researchers’ attention to alternative redox couples^54^. Great advances have been made recently, resulting in the implementation of Co-, Fe-, and Cu-based inorganic redox couples and thiolate-based organic redox couples^14, 41, 55–67^. Interest in Co-based complexes as an alternative redox shuttle in DSSCs has been increasing rapidly owing to their negligible visible light absorption and lower corrosiveness^55–60, 66, 67^. In 2009, an impressive open-circuit photovoltage of 0.35 V was obtained for the p-DSSC using Co^II/III^ tris(4,4′-di-*tert*-butyl-2,2′-dipyridyl) as electrolyte, giving an efficiency of 0.2%. The efficiency of the corresponding tandem device was 0.55%^57^. In 2011, members of the same group reported series of cobalt polypyridyl-based complexes and PCE of the p-DSSCs ranged from 0.04 to 0.24%^56^. In 2013, Powar *et al*. reported a novel electrolyte based on Co^II/III^ tris(1,2-diaminoethane) for p-DSSCs, the best cell yielded an efficiency of 1.3%^59^.

**Figure 2:**
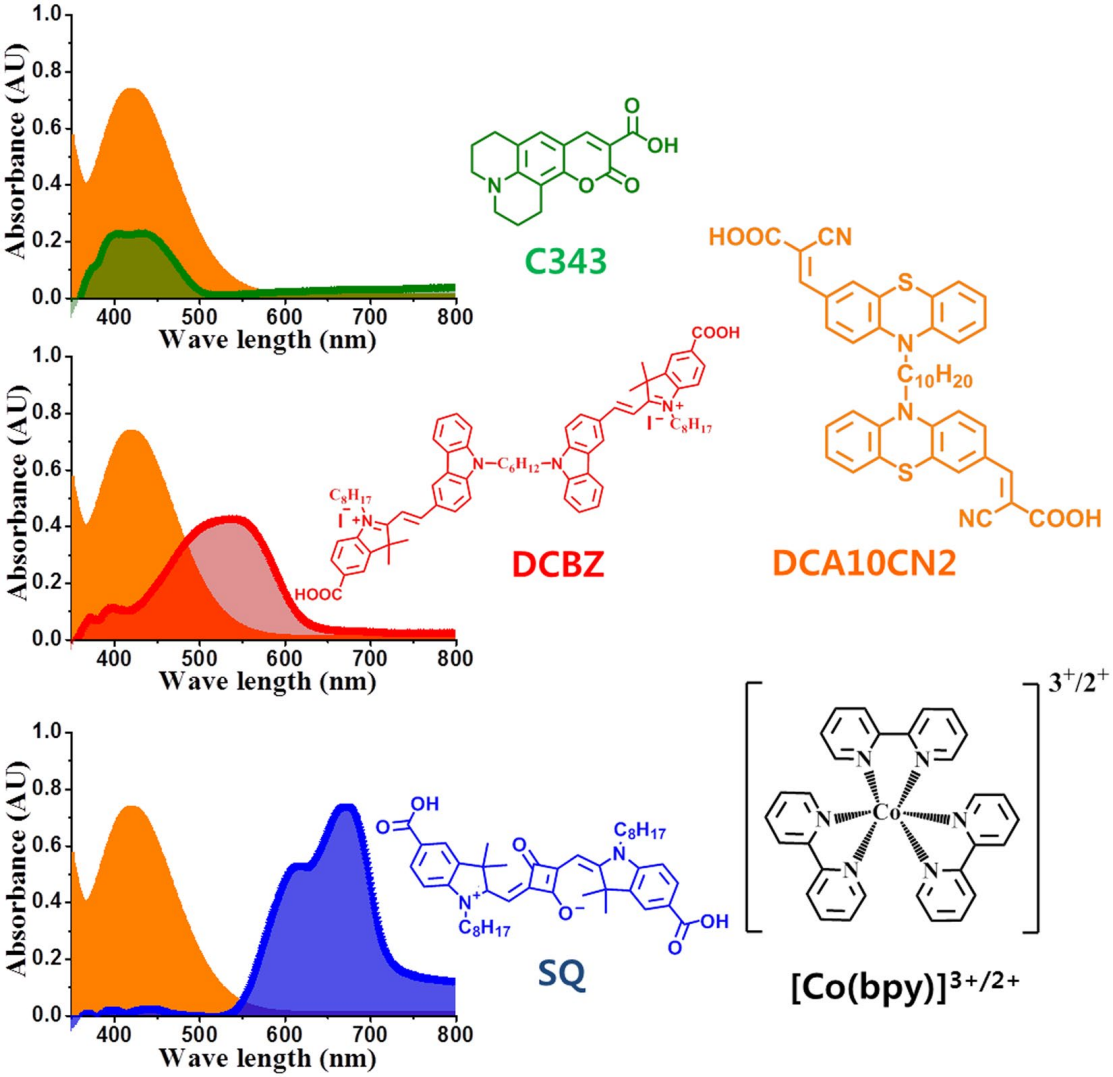
Pictorial representation of complementary absorption spectra of the dyes used in this study and chemical structures. Absorption spectrum of n-type DCA10CN2 overlapped with those of C343, DCBZ, and SQ p-type dyes with molecular structure along with molecular structure of cobalt complex.

Achieving high-PCE pn-DSSCs entails the development of (i) novel photocathodes with a higher ionisation potential to improve the photovoltage, (ii) optically transparent and non-corrosive electrolytes, and (iii) sensitisers with complementary absorption spectra. The aim of this work is to address (ii) and (iii). To address requirement (ii), we use an optically transparent and less corrosive Co^II/III^ tris(2,2′-dipyridyl) electrolyte. To address requirement (iii), we study the effect of the optical and electronic energy levels of the p-type component on the photovoltaic performance of tandem pn-DSSCs. Using p-type dye sensitisers with different absorption maxima yields dyes that absorb longer wavelengths to complement the photoanode sensitiser in tandem devices (Fig. 2). Among photocathodes fabricated using three well known dyes, C343^18–27^, DCBZ^16, 26^, and SQ^16, 68^, an SQ-based photocathode yielded near-IR absorption by the photocathode and complementary absorption between the photoanode and photocathode in pn-DSSCs (Fig. 2).

For the photoanode dye, based on above discussed double branched dye concept, we selected DCA10CN2 (Fig. 1), a nonconjugated bridged double-branched organic dye designed to transfer electron density over longer diffusion lengths^34^. The resulting Co-based tandem devices exhibited a promising efficiency of 1.41% with a short-circuit current density (*J*_SC_) of 4.00 mA cm^−2^, an open-circuit voltage (*V*_OC_) of 0.70 V, and a fill factor (FF) of 50.44%, which is five times higher than that of a device with an I^−^/I_3_^−^ redox mediator (0.26%). However, for devices with a Co redox electrolyte, a mismatch in the highest occupied molecular orbital (HOMO) level resulted in the lowest conversion efficiency for DCBZ dye, although its light harvesting ability is better than that of C343.

## Results and Discussion

### Optical properties

**Figure 3:**
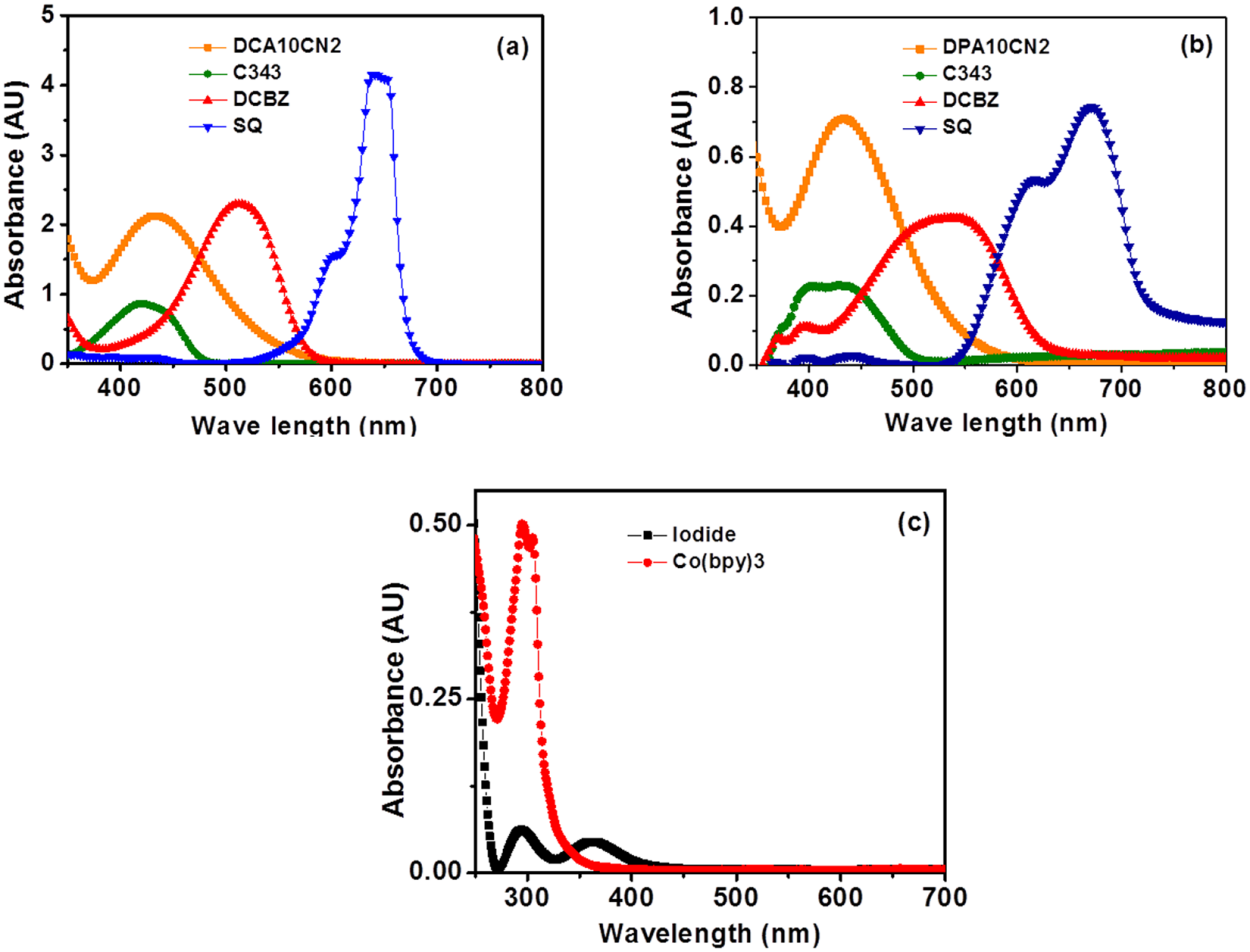
UV–visible absorption spectra. UV–visible absorption spectra of the dyes DCA10CN2, C343, DCBZ, and SQ (**a**) in solution and (**b**) adsorbed on photoelectrode films. (**c**) Absorption spectra of iodine and cobalt electrolytes.

The absorption spectra of DCA10CN2, C343, DCBZ, and SQ dyes measured in dimethylformamide and on films are depicted in Fig. 3a and b, respectively, and the numerical data are summarised in Table 1. The aim of this work was to study the effect of the optical and electronic energy levels of the p-type component on the photovoltaic performance of the tandem pn-DSSCs. Table 1 provides the optical and electrochemical data of the corresponding dyes. Using p-type dye sensitisers with different absorption maxima yields dyes that absorb longer wavelengths that complement those of the state-of-the-art photoanodes in tandem cells (Figs 1 and 2). For the photoanode dye, we synthesised DCA10CN2 (Fig. 2), a double-branched organic dye designed to transfer electron density toward the n-type semiconductor via the carboxylic acid anchoring group. Conversely, C343 pulls electrons away from the semiconductor surface and is one of the best-performing dyes with NiO. However, the spectral response of C343 almost entirely overlaps that of DCA10CN2 (Fig. 2). To avoid this problem, we have selected another two dyes, DCBZ and SQ, which have longer absorption wavelengths than C343. Changing the absorption maxima of the dyes to a lower energy is expected to improve the light harvesting ability by increasing the photocurrent density from the photocathode side. Among the cells using each dye, the SQ-based cell showed longer-wavelength absorption, which is spectrally complementary to that of the n-type sensitiser DCA10CN2, and exhibited the highest PCE (Fig. 3). From the onset absorption spectra, *E*_0–0_ values of 2.59, 2.16, and 1.85 eV were extracted for C343, DCBZ, and SQ dyes, respectively. In addition, Fig. 3c shows the UV–vis spectra of the electrolytes employed in this study, which are in agreement with the literature^34^. The electrolyte is lighter in colour than the iodine-based redox couple, which shows strong visible light absorption up to more than 450 nm. The negligible absorption of the cobalt electrolyte indicates that competition with the light absorption of the molecular dye sensitiser below the 500 nm region could be avoided, improving the photocurrent of the DSSCs.

**Table 1:** Optical and electrochemical data for dyes used in this study.

Dye	*λ*_*max*_ (nm) Solution TiO_2_	E_HOMO_ (V)	E^*^_LUMO_ (V)	E_0–0_ (eV)	ΔG_inj_ (eV)	ΔG_reg_ (eV)
DCA10CN2	401 420	—	—	—	—	—
C343	418 418	1.20	−1.39	2.59	−0.66	−1.95
DCBZ	511 528	0.64	−1.52	2.16	−0.10	−2.08
SQ	641 670	0.93	−0.92	1.85	−0.39	−1.48

### Photovoltaic properties

**Figure 4:**
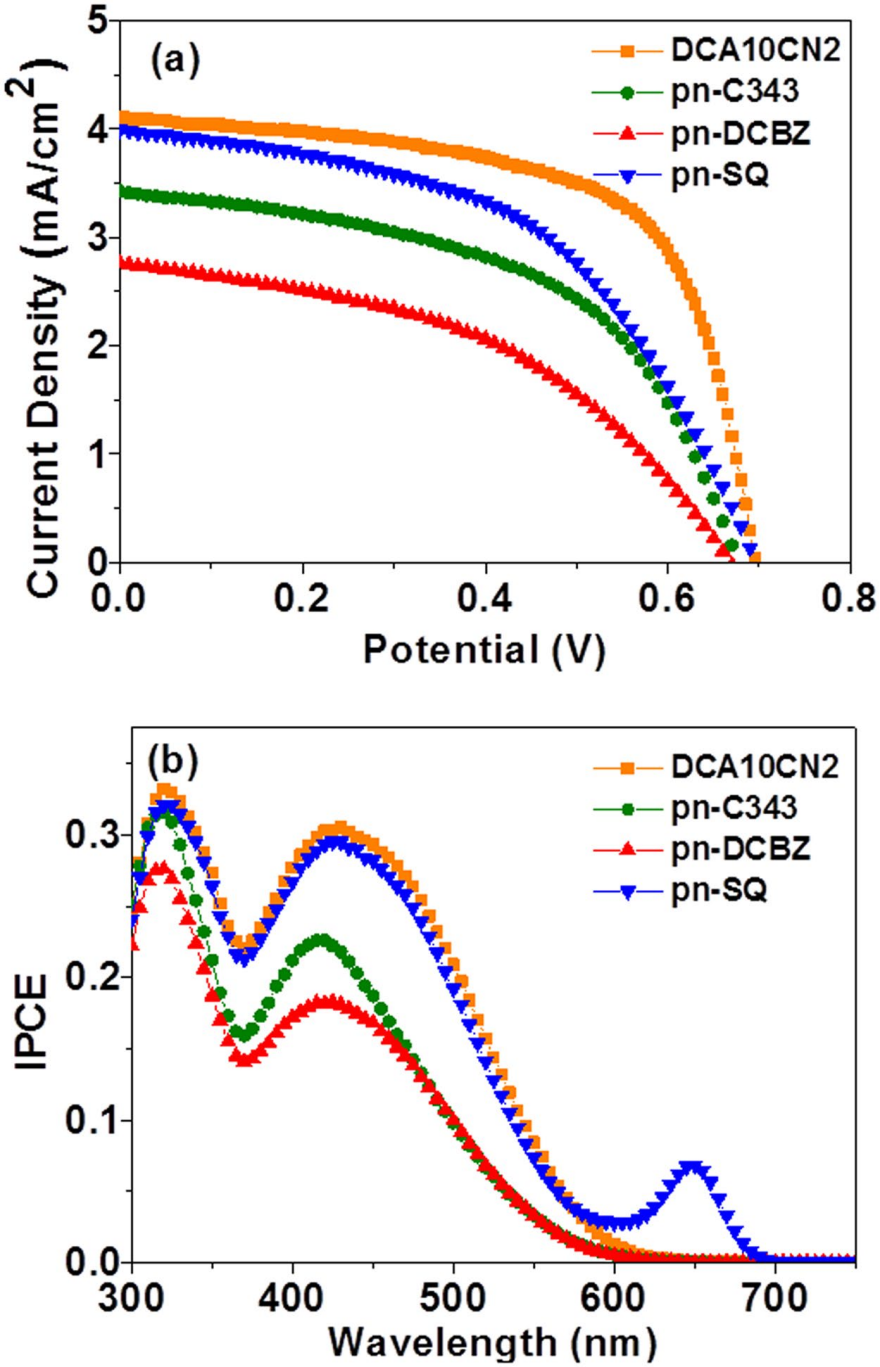
Photovoltaic characterization of pn-DSSCs illuminated through the n-side under AM 1.5 conditions (100 mW cm^−2^). (**a**) Current density-voltage (J-V) characteristics and (**b**) incident photon-to-current conversion efficiency spectra (IPCE).

Two electrolyte solutions were employed; the first, I^−^/I_3_^−^, consisted of 0.6 M DMPII, 0.05 M I_2_, and 0.5 M *t*BP in acetonitrile, and the second, Co(bpy)_3_^2+/3+^, consisted of 0.2 M [Co(bpy)_3_](PF_6_)_2_, 0.02 M [Co(bpy)_3_](PF_6_)_3_, 0.1 M LiClO_4_, and 0.5 M *t*BP in acetonitrile. Figures 4 and Supplementary Information, Fig. S1(a) show the photocurrent density–photovoltage (*J*–*V*) curves of the pn-DSSCs sensitised with a set of p-type dyes with different absorption wavelengths and using the Co- based and I^−^/I_3_^−^ redox couples, respectively, under standard global conditions through the n-side of the pn-DSSC. Table 1 show the photovoltaic parameters of the devices using the Co-based electrolytes illuminated through n-side. The *J*–*V* curves of the pn-DSCs illuminated through the p-side are presented in Supplementary Information Fig. S1(b and d). Supplementary Information, Tables S1 and S2 show the photovoltaic parameters of the devices using the Co-based and I^−^/I_3_^−^ electrolytes, respectively, under standard global conditions through the n- or p-side of the pn-DSSC; for comparison, results of devices illuminated through the n-side are also included. The error bars in Tables 1, S1 and S2 were calculated from the J–V curves of three devices for each condition. The considerable change in the photovoltaic performance of the devices when the light was illuminated through different sides (n-side or p-side) is a result of the change in current density values. This could be due the considerable difference of incoming photons at the n-side. The absorptivity of the Co-based redox couples typically remains well below 10% over most of the wavelength regime and these are optically dilute compared to iodine-based electrolyte, accomplishing higher number of photons available for the dye molecules. Powar *et al*. showed that for Co-based devices, the front and corrected back induced-photon-to-current conversion efficiencies (IPCEs) agree closely across the entire spectrum, whereas the corrected back IPCE spectra of I^−^/I_3_^−^-based devices drop regularly to reach a value close to 0% at about 380 nm^59^. This feature is an attractive option for pn-DSSCs, where one of the two dye-coated electrodes needs to run in a rare-illumination mode. Thus, we obtained excellent current densities by employing the Co-based redox couple, obtaining higher PCEs in the range of 0.8–1.41% (Fig. 4 and Table 2) under standard global conditions, whereas the iodine-based devices showed lower PCEs of 0.08–0.5% (Supplementary Information, Fig. S1 and Table S2) under similar measurement and fabrication conditions. Note that the photoelectrode film (TiO_2_ and NiO) thicknesses for both of these cells were optimised for pn devices using a Co-based redox couple.

**Table 2:** Photovoltaic performance of 3 cells of each type of n-DSSC and pn-DSSCs (along with their standard deviations) illuminated through n-side under AM 1.5 G conditions based on DCA10CN2, C343, DCBZ, and SQ dyes using Co^+2/+3^ electrolyte.

Device	J_SC_ (mA/cm^2^)	V_OC_ (V)	FF (%)	η (%)
n-DSSC	4.109 ± 0.355	0.696 ± 0.005	63.78 ± 0.18	1.824 ± 0.165
pn-DSSC/C343	3.427 ± 0.116	0.677 ± 0.009	52.76 ± 1.13	1.224 ± 0.034
pn-DSSC/DCBZ	2.763 ± 0.102	0.668 ± 0.001	45.25 ± 0.91	0.835 ± 0.018
pn-DSSC/SQ	3.966 ± 0.069	0.697 ± 0.002	50.45 ± 1.39	1.405 ± 0.005

When the Co-based electrolyte was employed, the open-circuit voltages (*V*_OC_) and FFs of the three cells were more or less similar to each other, whereas the short-circuit current densities (*J*_SC_) differed. *J*_SC_ decreased in the order SQ (4.00 mA cm^−2^) > C343 (3.43 mA cm^−2^) > DCBZ (2.76 mA cm^−2^). Consequently, the PCE also decreased in the order SQ (1.41%) > C343 (1.22%) > DCBZ (0.84%). The redward shift of the absorption pattern of the SQ dye resulted in an increased *J*_SC_, leading to a higher PCE. DCBZ exhibited lower photovoltaic performance than C343, despite its longer absorption range than C343, as shown in Fig. 3. This could be due to the mismatched energy level of the corresponding dye, which will be discussed in the following section. The IPCE spectra of the tandem pn-DSSCs (Fig. 4b) are consistent with their *J*_SC_ values. The *J*_SC_ values calculated from the spectra were also more or less close to those listed in Table 1, indicating the accuracy of the measurements. In Fig. 4b, a shoulder peak clearly appears in the IPCE spectra of the tandem device at long wavelengths of 600–700 nm. It obviously originates from the SQ sensitiser adsorbed on the NiO photocathode, as seen in the UV–vis absorption spectrum (Fig. 3a). The IPCE studies unambiguously indicated that the SQ sensitiser on the photocathode contributed to current generation; that is, they confirmed the successful construction of the tandem pn-DSSC.

### Electrochemical properties

**Figure 5:**
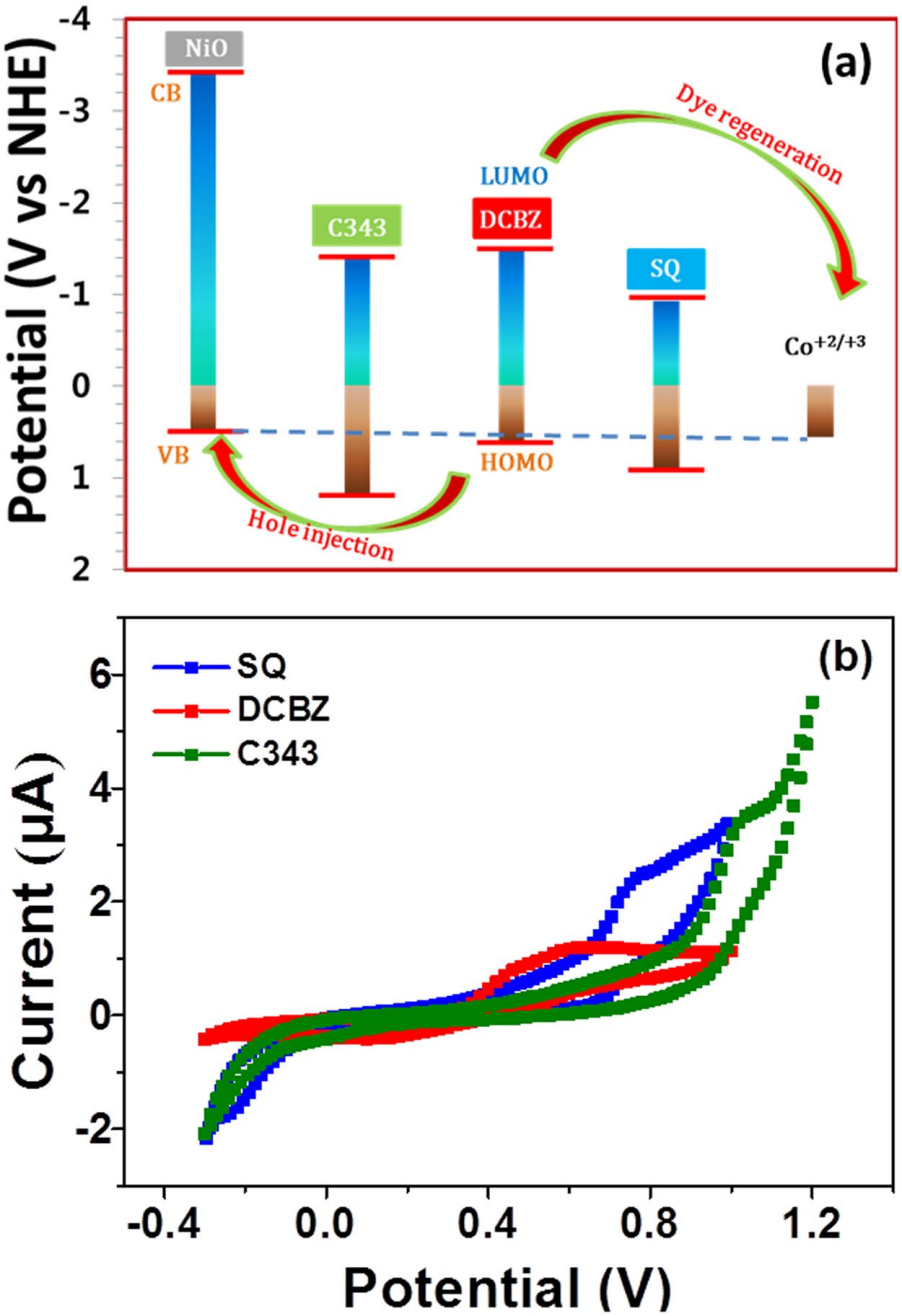
Energy levels and Cyclic Voltammograms. (**a**) Energy level diagram of each component in p-type photovoltaic device and (**b**) cyclic voltammograms of C343, DCBZ, and SQ obtained at a scan rate 100 mV s^−1^.

In every instance, the HOMO and lowest unoccupied molecular orbital (LUMO) of the dye molecule should provide a large enough driving force compared to the electrolyte potential and the valence band potential for the forward reactions to occur (Fig. 5a). Diminished device performance could be interpreted as the result of either i) dye-sensitised electron transfer to the conduction band of the NiO photocathode film or ii) interfacial hole recombination in the NiO valence band and the existence of redox species in the electrolyte. Figure 5a provides data on the HOMO and LUMO levels calculated from the cyclic voltammetry result (Fig. 5b) and the numerical data are summarised in Table 1. When the dye’s HOMO is situated between the energy level of the electrolyte and the upper valence band edge of NiO, hole injection from the excited state of the dye into the valence band of NiO is no longer possible. In this case, hole transfer from the semiconductor to the dye becomes energetically favoured in a situation that would result in strong recombination currents. Surprisingly, DCBZ dye, which had better spectral complementarity than C343 dye for tandem DSSCs, exhibits a HOMO level mismatch with the NiO valence band (Fig. 5a). In addition, the energy levels of the cobalt electrolyte, the dye’s HOMO level, and the NiO valence band edge fall on the same line (Fig. 5a). Because DCBZ dye is described by ii) above, hole injection from the excited state of the dye into the valence band of NiO is thermodynamically not feasible. Consequently, this phenomenon could result in strong recombination currents, degrading the photovoltaic performance of DCBZ dye. The hole injection driving force (Δ*G*_inj_) can be determined according to the equation Δ*G*_inj_ = *e*{*E*_VB_(NiO) − [*E*_0–0_(S*) + *E*_red_(*S*/*S* ^−^)]}, where the valence band energy *E*_VB_(NiO) = 0.54 V vs. NHE^46^. The measured *E*_red_ values were −1.39, −1.52, and −0.92 V (vs. NHE) for C343, DCBZ, and SQ, respectively. The hole injection driving forces were determined to be −0.66 eV for C343, −0.10 eV for DCBZ, and −0.39 eV for SQ. From these values, it is clear that DCBZ dye could not pull electrons from NiO following light absorption. In the p-type DSSCs, the reduced sensitisers were oxidised by the electrolyte. The redox potential of *E*(Co^+2/+3^) is 0.56 V vs. NHE, which is more positive than the *E*_red_(*S*/*S* ^−^) level of the dyes used for the photocathode (C343, DCBZ, and SQ). This difference will afford a thermodynamically favourable driving force for effective regeneration of dyes in the p-type DSSCs^69, 70^. The dye regeneration driving forces can be determined according to the equation Δ*G*_reg_ = *e*[*E*_red_(*S*/*S* ^−^) − *E*(*M*/*M* ^−^)]^69^. The dye regeneration driving forces were determined to be −1.95 eV for C343, −2.08 eV for DCBZ, and −1.48 eV for SQ. Except for the lower Δ*G*_inj_ value for the DCBZ dye, the charge transfer processes are exothermic, and the driving forces satisfy the requirement for efficient charge transfer. Therefore, the lower photocurrent and photovoltaic performance of the DCBZ dye-sensitised device were ascribed to the lower Δ*G*_inj_ value^69^.

Our results indicate that as the absorption maximum of the dye is increased, the light harvesting capability of the sensitiser is enhanced. The largest *J*_SC_ is realised when SQ is used as the sensitiser. The lowest *J*_SC_, however, is obtained when DCBZ is used as the sensitiser. These results suggest that the lowest *J*_SC_ value of DCBZ is likely caused by the HOMO level mismatch with the NiO valence band. This mismatched HOMO energy level facilitates strong recombination currents. Changing the dye’s molecular structure further by optimising the energy levels will allow us to improve *J*_SC_ even further. However, the primary requirements for improving the PCE are the adoption of NiO as the photocathode material and exploring photocathodes with an energetically lower-lying valence band.

## Conclusions

In an attempt to improve the efficiency of DSSCs, we investigated tandem pn-DSSCs using an optically dilute and less-corrosive cobalt-based redox electrolyte. We investigated several combinations of various dyes with different spectral complementarity in tandem pn-DSSCs and studied how the dye structure affects the optical and electrochemical properties of the cells. We found that when the absorption maxima of the p-type dye molecules shifted toward the red region, the photocurrent and efficiency of the pn-DSSCs increased. However, the photocurrent and efficiency decreased when DCBZ dye was used despite its better complementarity than C343 dye. A basic electrochemical study showed that this difference stemmed from a mismatched HOMO level, which resulted in a smaller hole injection driving force. The best PCE of 1.41% (*J*_SC_ = 4.00 mA cm^−2^, *V*_OC_ = 0.70 V, and FF = 50.44%) was obtained for a series-connected pn-DSSC containing DCA10CN2 as the photoanode dye and SQ as the photocathode dye. The basic results are promising, and further studies should focus on developing novel dye sensitisers yielding complementary absorption with the potential to generate larger photocurrents, which can enhance the device efficiency even further.

## Methods

### Materials and characterisation

All solvents and chemicals were received from commercial sources and used without further purification. Terpineol, ethyl cellulose, ethanol, acetonitrile, hydrogen peroxide, 2,2′-bipyridine, lithium perchlorate (LiClO_4_), cobalt(II) chloride hexahydrate (CoCl_2_·6H_2_O), and 4-*tert*-butylpyridine (*t*BP) were purchased from Sigma-Aldrich. Nickel oxide nanopowder (NiO, 20 nm in size) was purchased from Inframat. Chloroplatinic acid hexahydrate (H_2_PtCl_6_) and TiO_2_ paste (18-NRT) were purchased from Dyesol. Fluorine-doped tin oxide (FTO) glass substrates with a sheet resistance of 8 Ω cm^−2^ were obtained from PilkingtonThe dye sensitisers DCA10CN2, C343, DCBZ, and SQ were prepared following previous reports^26, 34, 68^. The cobalt complexes, [Co(bpy)_3_](PF_6_)_2_ and [Co(bpy)_3_](PF_6_)_3_ (bpy = 2,2′-bipyridine), were synthesised according to our previously published procedure^58^. The absoption speca were recorded using an Agilent 845X UV-vis/near infrared spectrophotometer. The photovoltaic current density-voltage characterisation was performed under 1 sun AM 1.5 G simulated sunlight (PEC-L11, Peccell Technologies, Inc.). The monochromatic IPCEs were plotted as a function of the wavelength using an IPCE measurement instrument (PEC-S20, Peccell Technologies, Inc.). The active areas of the dye-sensitised films were estimated using a digital microscope camera with image-analysis-software (Moticam 1000). The thickness of the photoelectrodes was 4 μm, as determined using a profilometer (Surfcorder ET-3000, Kosaka Laboratory Ltd.). The redox properties of dyes were studied by using cyclic voltammetry (Model: CV-BAS-Epsilon). The electrolyte solution used was 0.1 M tetrabutylammonium tetrafluoroborate (TBA(BF_4_)) in dried acetonitrile. The Pt and Ag/AgCl wire electrodes were used as counter and reference electrodes, respectively.

### Device fabrication

The FTO glass film substrates were washed with deionised water, ethanol, and acetone using an ultrasonic bath for 20 min. The NiO paste was prepared as reported elsewhere^12^: a slurry of 6 g of NiO in a solution of ethanol was mixed with 20 mL of 10 wt.% ethanolic ethyl cellulose solution and 40 mL of terpineol, and then the ethanol was removed using a rotary vacuum evaporator. The mesoporous NiO layer was deposited on the clean FTO using a screen-printing technique. Then the films were dried at 70 °C for 45 min in air and sintered at 450 °C for 30 min. The resulting films were dipped in a 0.1 mM solution of C343, DCBZ, or SQ dye in dry acetonitrile for 48 h at 25 °C for dye absorption. For the photoanode, mesoporous TiO_2_ films were assembled by a similar process^16^ using a commercial paste (18-NRT, Dyesol). The screen-printed and sintered TiO_2_ films were immersed in a 0.3 mM solution of DCA10CN2 dye in ethanol and held at 25 °C for 24 h.

For both n-type and p-type devices, Pt counter electrodes were prepared by applying a single drop of a H_2_PtCl_6_ solution (7 mM in isopropanol) to clean the FTO glass, followed by thermal decomposition by annealing at 450 °C for 30 min, similar to the previous report. The single-junction device was assembled by arranging face-to-face a Pt-coated FTO substrate and a dye-sensitised working electrode using a thick Surlyn resin (60 μm; DuPont 1702). The tandem devices were assembled by sandwiching a p-type photocathode and an n-type photoanode. The devices were then heated to 120 °C for 1 min to form a seal. The iodine-based electrolyte consisted of 0.6 M 1,2-dimethyl-3-propylimidazolium iodide (DMPII), 0.05 M I_2_, and 0.5 M *t*BP in acetonitrile. The cobalt-based electrolyte consists of 0.2 M [Co(bpy)_3_](PF_6_)_2_, 0.02 M [Co(bpy)_3_](PF_6_)_3_, 0.1 M LiClO_4_ and 0.5 M *t*BP in acetonitrile. The required electrolyte was injected into the gap between the two electrodes. The chemical structure of the Co complex is shown in Fig. 2.

## Electronic supplementary material

Supplementary Information
