# Supplementary material for: Discrete photoelectrodes with dyes having different absorption wavelengths for efficient cobalt-based tandem dye-sensitised solar cells
Source: Sci Rep. 2017 May 23;7:2272. doi: 10.1038/s41598-017-02480-y (PMC5442119; doi:10.1038/s41598-017-02480-y)
Supplement: Supplementary file 1 — Supplementary Information [file 41598_2017_2480_MOESM1_ESM.pdf]

Supporting information

Discrete photoelectrodes with dyes having different absorption wavelengths for efficient cobalt-based tandem dye-sensitised solar cells

Phuong Ho, Suresh Thogiti*, Yong Hui Lee, Jae Hong Kim*

School of Chemical Engineering, Yeungnam University, 214-1, Dae-dong, Gyeongsan-si, Gyeongsangbuk-do 712-749, Republic of Korea.

*Email: sureshyu@ynu.ac.kr; jaehkim@ynu.ac.kr

TEL : +82-53-810-2521

FAX : +82-53-810-4631

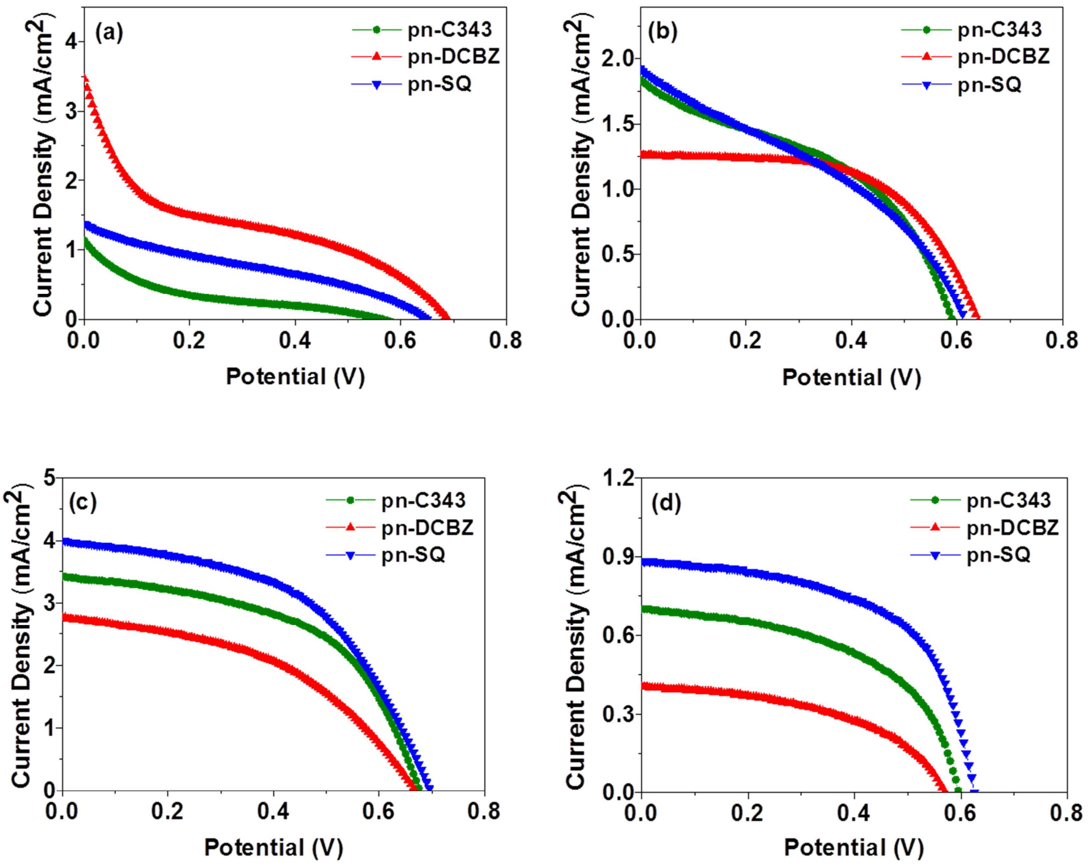

Supplementary Figure S1: Current density–voltage (*J–V*) curves of the pn-DSSCs sensitised with a set of p-type dyes with different absorption wavelengths under standard global conditions. (a) device illuminated through n-side using I^-/I_3^- based electrolyte, (b) device illuminated through p-side using I^-/I_3^- based electrolyte, (c) device illuminated through n-side using $Co^{+2/+3}$— based electrolyte, (d) device illuminated through p-side using Co— based electrolyte.

Supplementary Table S1: Photovoltaic performance of 3 cells of each type of n-DSSC and pn-DSSCs (along with their standard deviations) based on DCA10CN2, C343, DCBZ, and SQ using $Co^{+2/+3}$ electrolyte.

Device	Illumination	J_{SC} (mA/cm^2)	V_{OC} (V)	FF (%)	η (%)
**n-DSSC**	n-side	4.109 ± 0.355	0.696 ± 0.005	63.78 ± 0.18	1.824 ± 0.165
**pn-DSSC/C343**	n-side	3.427 ± 0.116	0.677 ± 0.009	52.76 ± 1.13	1.224 ± 0.034
	p-side	0.702 ± 0.015	0.596 ± 0.001	51.44 ± 0.39	0.215 ± 0.006
**pn-DSSC/DCBZ**	n-side	2.763 ± 0.102	0.668 ± 0.001	45.25 ± 0.91	0.835 ± 0.018
	p-side	0.404 ± 0.045	0.570 ± 0.015	47.50 ± 2.47	0.109 ± 0.019
**pn-DSSC/SQ**	n-side	3.966 ± 0.069	0.697 ± 0.002	50.45 ± 1.39	1.405 ± 0.005
	p-side	0.884 ± 0.053	0.625 ± 0.003	57.12 ± 2.31	0.316 ± 0.033

Supplementary Table S2: Photovoltaic performance of 3 cells of each type of n-DSSC and pn-DSSCs (along with their standard deviations) based on DCA10CN2, C343, DCBZ, and SQ using I^{-}/I_3^{-} electrolyte.

Device	Illumination	J_{SC} (mA/cm^2)	V_{OC} (mV)	FF (%)	η (%)
**n-DSSC**	n-side	11.53 ± 0.263	0.574 ± 0.006	51.51 ± 1.52	3.409 ± 0.015
**pn-DSSC/C343**	n-side	1.127 ± 0.068	0.565 ± 0	12.41 ± 1.19	0.079 ± 0.003
	p-side	1.842 ± 0.010	0.590 ± 0.003	41.58 ± 1.39	0.452 ± 0.016
**pn-DSSC/DCBZ**	n-side	3.462 ± 0.002	0.685 ± 0.005	21.10 ± 0.09	0.500 ± 0.005
	p-side	1.262 ± 0.029	0.639 ± 0.010	57.96 ± 0.29	0.467 ± 0.021
**pn-DSSC/SQ**	n-side	1.369± 0	0.649 ± 0.011	29.70 ± 0.05	0.264 ± 0.005
	p-side	1.927 ± 0.025	0.610 ± 0.005	35.32 ± 0.07	0.415 ± 0.001
